# Structural and biochemical analyses of monoubiquitinated human histones H2B and H4

**DOI:** 10.1098/rsob.160090

**Published:** 2016-06-22

**Authors:** Shinichi Machida, Satoshi Sekine, Yuuki Nishiyama, Naoki Horikoshi, Hitoshi Kurumizaka

**Affiliations:** 1 Laboratory of Structural Biology, Graduate School of Advanced Science and Engineering, Waseda University, 2-2 Wakamatsu-cho, Shinjuku-ku, Tokyo 162-8480, Japan; 2 Research Institute for Science and Engineering, Waseda University, 2-2 Wakamatsu-cho, Shinjuku-ku, Tokyo 162-8480, Japan; 3 Institute for Medical-oriented Structural Biology, Waseda University, 2-2 Wakamatsu-cho, Shinjuku-ku, Tokyo 162-8480, Japan

**Keywords:** histone, ubiquitin, nucleosome, chromatin, crystal structure

## Abstract

Monoubiquitination is a major histone post-translational modification. In humans, the histone H2B K120 and histone H4 K31 residues are monoubiquitinated and may form transcriptionally active chromatin. In this study, we reconstituted nucleosomes containing H2B monoubiquitinated at position 120 (H2Bub_120_) and/or H4 monoubiquitinated at position 31 (H4ub_31_). We found that the H2Bub_120_ and H4ub_31_ monoubiquitinations differently affect nucleosome stability: the H2Bub_120_ monoubiquitination enhances the H2A–H2B association with the nucleosome, while the H4ub_31_ monoubiquitination decreases the H3–H4 stability in the nucleosome, when compared with the unmodified nucleosome. The H2Bub_120_ and H4ub_31_ monoubiquitinations both antagonize the Mg^2+^-dependent compaction of a poly-nucleosome, suggesting that these monoubiquitinations maintain more relaxed conformations of chromatin. In the crystal structure, the H2Bub_120_ and H4ub_31_ monoubiquitinations do not change the structure of the nucleosome core particle and the ubiquitin molecules were flexibly disordered in the H2Bub_120_/H4ub_31_ nucleosome structure. These results revealed the differences and similarities of the H2Bub_120_ and H4ub_31_ monoubiquitinations at the mono- and poly-nucleosome levels and provide novel information to clarify the roles of monoubiquitination in chromatin.

## 1. Introduction

In eukaryotes, genomic DNA is folded into a higher-order structure called chromatin and is accommodated within the nucleus [1]. The nucleosome is the basic repeating unit of chromatin, and histone proteins are highly conserved components. In the nucleosome, four core histones, H2A, H2B, H3 and H4, specifically form the H2A–H2B and H3–H4 heterodimeric complexes with histone-fold domains and two each of the H2A–H2B and H3–H4 dimers constitute the histone octamer [2]. The 145–147 base-pair DNA segments are left-handedly wrapped by about 1.7 turns around the histone octamer in the nucleosome [3–5].

The activities of genomic DNA, such as transcription, must be regulated in chromatin [6]. However, the DNA is generally inaccessible in chromatin, and therefore the DNA-binding proteins functioning in transcription must overcome the chromatin barrier [7–10]. Numerous histone modifications and histone variants contribute to the structural and physical versatility of nucleosomes and affect the chromatin dynamics, and thus play a crucial role to accomplish the regulation of genomic DNA in chromatin [10–21].

Acylation, methylation and phosphorylation are well-known chemical modifications of histones [11–15]. In addition, the covalent attachment of a small protein, ubiquitin, has also been identified as a histone lysine modification [22]. These histone modifications are considered to function in organizing the chromatin domains, such as transcriptionally active euchromatin and inactive heterochromatin. For example, histone acetylation is commonly detected in euchromatic regions, and histone methylations, such as the H3 K9 and K27 methylations, are predominantly found in heterochromatic regions [12–14]. Similarly, for histone ubiquitination, histone H2B K120 (K123 for budding yeast) monoubiquitination is present in transcriptionally active genes [23–25]. Histone H4 K31 monoubiquitination is reportedly also associated with active chromatin regions [26]. By contrast, histone H2A monoubiquitination is mainly found in facultative heterochromatin, such as the inactive X chromosome, and in regions containing silenced genes [27–30]. Therefore, the monoubiquitination of individual histones at certain amino acid residues may have a distinct function and probably affects the structure and physical properties of the nucleosome. Accordingly, H2B K120 monoubiquitination reportedly inhibits the compaction of poly-nucleosomes *in vitro* [31]. However, the molecular mechanism by which the ubiquitin molecule affects the chromatin conformation has not been clarified yet.

In this study, we prepared human histones H2B and H4, in which ubiquitin molecules were chemically conjugated at H2B-120 and H4-31, respectively. We then performed biochemical and structural analyses of nucleosomes and poly-nucleosomes containing these monoubiquitinated histones H2B and H4 *in vitro*.

## 2. Results

### 2.1. Reconstitution of nucleosomes containing monoubiquitinated histones H2B and H4

To study the effect of the histone monoubiquitination on the characteristics of the nucleosome, we prepared histones H2B and H4 that were monoubiquitinated at positions 120 and 31, respectively. To do so, the H2B K120C and H4 K31C mutants, in which the H2B K120 and H4 K31 residues were replaced by cysteine, respectively, were purified as recombinant proteins, and the ubiquitin molecule was chemically conjugated by a disulfide bond to the H2B C120 and H4 C31 residues [32]. In this study, the H2B and H4 proteins that were monoubiquitinated at the 120 and 31 positions by this method were named H2Bub_120_ and H4ub_31_, respectively.

**Figure 1.**
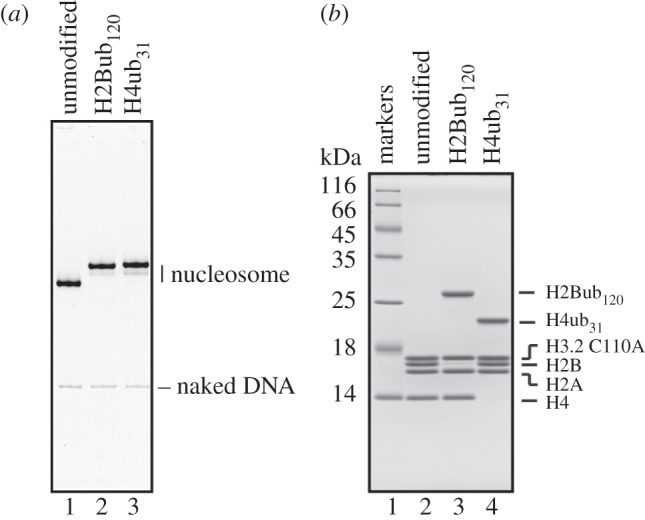
Reconstitution of the H2Bub_120_ and H4ub_31_ nucleosomes. (*a*) Purified H2Bub_120_ and H4ub_31_ nucleosomes were analysed by 6% native-PAGE with EtBr staining. Lanes 1–3 indicate the unmodified, H2Bub_120_ and H4ub_31_ nucleosomes, respectively. (*b*) Histone compositions of the purified H2Bub_120_ and H4ub_31_ nucleosomes, analysed by 18% SDS-PAGE with Coomassie Brilliant Blue staining. Lane 1 indicates molecular mass markers and lanes 2–4 represent the unmodified, H2Bub_120_ and H4ub_31_ nucleosomes, respectively.

We then reconstituted the nucleosomes containing H2Bub_120_ or H4ub_31_ and purified them by preparative native polyacrylamide gel electrophoresis. The purified H2Bub_120_ and H4ub_31_ nucleosomes migrated slowly on the native polyacrylamide gel, when compared with the unmodified nucleosome, due to the two conjugated ubiquitin molecules (figure 1*a*). An analysis by SDS-polyacrylamide gel electrophoresis revealed that the monoubiquitinated H2B and H4 were stoichiometrically incorporated into the nucleosomes, and only trace amounts of ubiquitin-free H2B and H4 were detected (figure 1*b*). These results indicated that the H2Bub_120_ and H4ub_31_ molecules were properly assembled into the nucleosomes.

### 2.2. Monoubiquitinations of H2B and H4 differently affect the nucleosome stability

**Figure 2.**
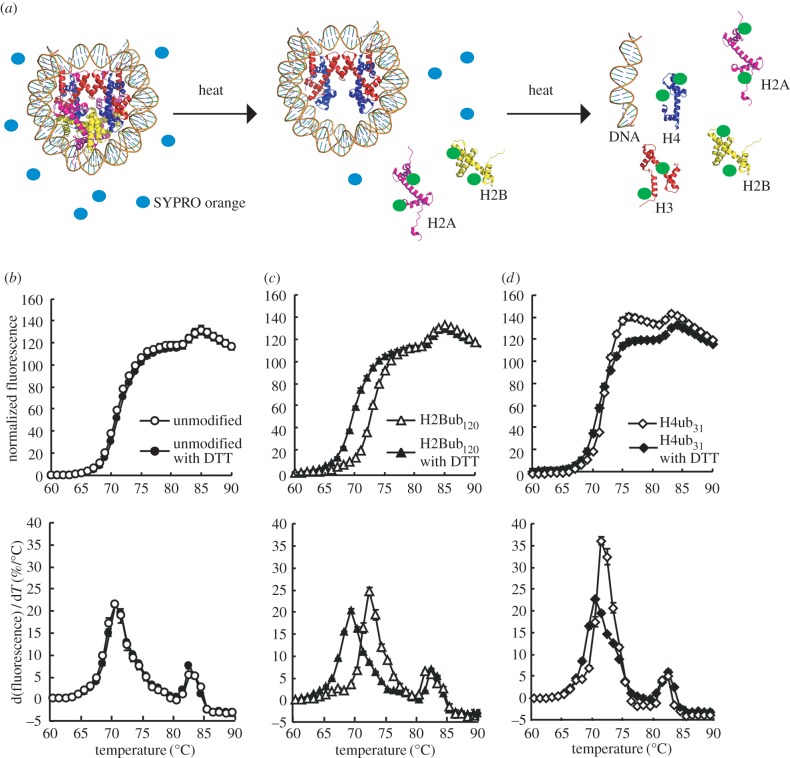
Stabilities of the H2Bub_120_ and H4ub_31_ nucleosomes. (*a*) Schematic diagram of the thermal stability assay. Histones H2A, H2B, H3 and H4 are coloured magenta, yellow, red and blue, respectively. Blue and bright green circles represent SYPRO Orange-free histones and SYPRO Orange-bound denatured histones, respectively. (*b*) Thermal stability curves of the unmodified nucleosomes in the presence (filled circles) or absence (open circles) of 10 mM dithiothreitol. (*c*) Thermal stability curves of the H2Bub_120_ nucleosomes in the presence (filled triangles) or absence (open triangles) of 10 mM dithiothreitol. (*d*) Thermal stability curves of the H4ub_31_ nucleosomes in the presence (filled diamonds) or absence (open diamonds) of 10 mM dithiothreitol. The normalized fluorescence intensities were plotted against each temperature from 60°C to 90°C. Means ± s.d. (*n* = 3) are shown. The derivative values of each stability curve are represented in (*b*–*d*), with standard deviations (*n* = 3).

We then tested the stability of the H2Bub_120_ and H4ub_31_ nucleosomes by a thermal stability assay. In this assay, nucleosome disruption was monitored as the fluorescence signal of SYPRO Orange bound to thermally denatured histones, which are released from the nucleosome (figure 2*a*). Consistent with the previous results [33], the unmodified nucleosome was disrupted with a bi-phasic denaturation curve, in which the first peak (Tm = 70–71°C) and second peak (Tm = 82–83°C) corresponded to the dissociation phases for H2A–H2B and H3–H4 from the nucleosome, respectively (figure 2*b*).

In these samples, the ubiquitin molecule was easily detached by a reducing agent, such as dithiothreitol. We then performed the thermal stability assay under conditions with or without dithiothreitol. Under the monoubiquitinated conditions (without dithiothreitol), the first peak of the H2Bub_120_ nucleosome was substantially shifted towards a higher temperature (Tm = 72–73°C), when compared with the experiments under the deubiquitinated conditions (with dithiothreitol) (figure 2*c*). These results indicate that the H2B monoubiquitination at position 120 enhances the association of H2A–H2B with the nucleosome.

Interestingly, under the monoubiquitinated conditions (without dithiothreitol), the H4ub_31_ nucleosome exhibited a distinct thermal denaturation curve, in which the second peak slightly shifted toward a lower temperature, and the height of the first peak was drastically increased (figure 2*d*). This thermal denaturation profile of the H4ub nucleosome is very similar to that of a nucleosome with unstable H3–H4, such as a nucleosome with the centromere-specific H3, CENP-A [34]. Therefore, the H4 K31 monoubiquitination may destabilize the association of H3–H4 with the nucleosome. This characteristic thermal denaturation profile of the H4ub_31_ nucleosome disappeared in the presence of dithiothreitol (figure 2*d*), indicating that the H4 monoubiquitination at position 31 is actually responsible for decreasing the nucleosome stability, in contrast with the H2B monoubiquitination.

### 2.3. Crystal structure of the nucleosome containing H2B and H4 monoubiquitinations

**Figure 3.**
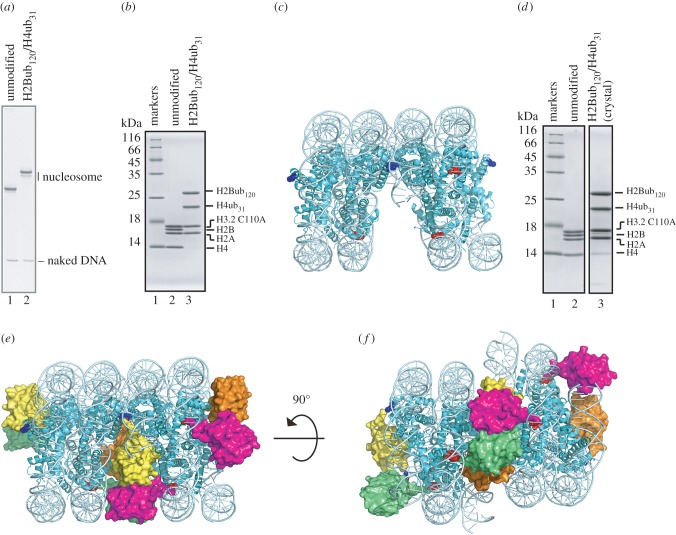
The crystal structure of the H2Bub_120_/H4ub_31_ nucleosome. (*a*) The purified H2Bub_120_/H4ub_31_ nucleosome was analysed by 6% native-PAGE with EtBr staining. Lanes 1 and 2 indicate the unmodified and H2Bub_120_/H4ub_31_ nucleosomes, respectively. (*b*) The purified H2Bub_120_/H4ub_31_ nucleosome was analysed by 18% SDS-PAGE with Coomassie Brilliant Blue staining. Lane 1 indicates molecular mass markers, and lanes 2 and 3 represent the unmodified and H2Bub_120_/H4ub_31_ nucleosomes, respectively. (*c*) The crystal structures of the H2Bub_120_/H4ub_31_ nucleosomes. Two neighbouring nucleosome molecules in the crystal are shown. The H2B C120 and H4 C31 residues are coloured blue and red, respectively. (*d*) The histone composition of the H2Bub_120_/H4ub_31_ nucleosome crystals was analysed by 18% SDS-PAGE with Coomassie Brilliant Blue staining. Lanes 1 and 2 indicate molecular mass markers and the unmodified nucleosome, respectively. Lane 3 represents the histone composition of the H2Bub_120_/H4ub_31_ nucleosome crystals. (*e*) The model structures of the H2Bub_120_/H4ub_31_ nucleosomes. The ubiquitin molecules (PDB: 1UBQ) are modelled in the H2Bub_120_/H4ub_31_ nucleosome structures shown in (*c*). The ubiquitin molecules attached to position 120 of H2Bs (blue spheres) of each nucleosome are coloured yellow and orange, and those attached to position 31 of H4s (red spheres) are coloured green and magenta, respectively. (*f*) An image of the H2Bub_120_/H4ub_31_ nucleosome models horizontally rotated by 90° relative to the image shown in (*e*).

We next studied whether the monoubiquitinations of H2B and H4 affect the nucleosome structure. We successfully reconstituted the nucleosome containing both H2Bub_120_ and H4ub_31_, and thus the H2B-K120 and H4-K31 monoubiquitinations are not mutually exclusive (figure 3*a*,*b*). We then determined the crystal structure of the nucleosome containing H2Bub_120_ and H4ub_31_ (the H2Bub_120_/H4ub_31_ nucleosome). In the crystal structure, the nucleosome core structure was not changed by the H2B and H4 monoubiquitinations, although the ubiquitin molecules were not visible (figure 3*c*). We confirmed that the ubiquitin molecules were not detached and were still covalently conjugated to the nucleosomal histones H2B and H4 in the crystals (figure 3*d*). In the crystals, the H2Bub_120_/H4ub_31_ nucleosomes contacted each other with a space that could accommodate four ubiquitin molecules (figure 3*e*,*f*). Therefore, the ubiquitin moieties conjugated to the nucleosomal H2B and H4 were located between the H2Bub_120_/H4ub_31_ nucleosomes in the crystal and were quite flexible.

Recently, the crystal structure of the H2Bub_120_ nucleosome bound to the deubiquitinating module of the SAGA complex (SAGA–DUB) was reported [35]. In the complex, SAGA–DUB directly bound to the acidic patch of the nucleosome surface and the ubiquitin molecule, but did not contact the nucleosome surface, except for its conjugation site. In the SAGA–DUB–nucleosome complex, the ubiquitin molecule was clearly visible, because its flexibility is restricted by binding to SAGA–DUB.

### 2.4. The H4 monoubiquitination at position 31 inhibits chromatin compaction, similar to the H2B monoubiquitination

**Figure 4.**
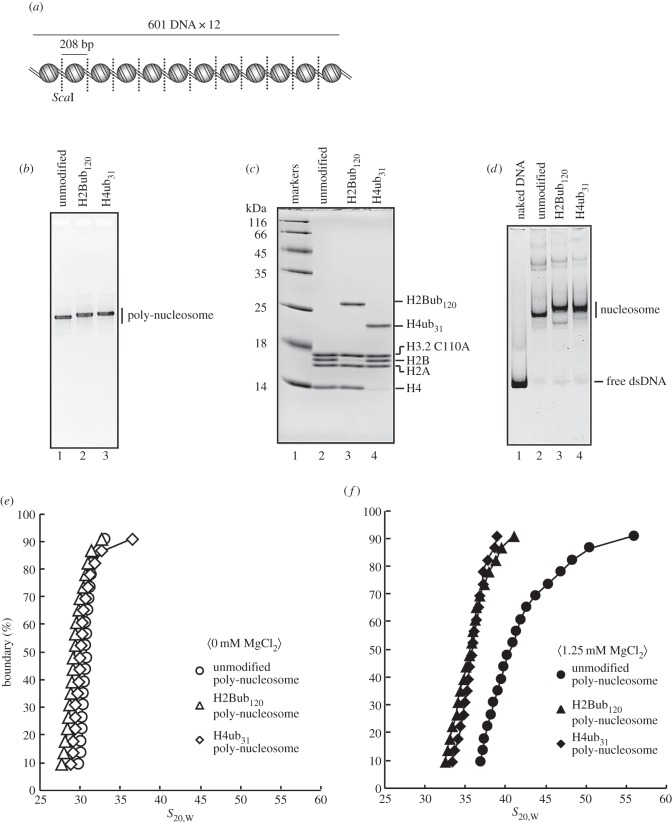
Sedimentation velocity analyses of poly-nucleosomes containing H2Bub_120_ and H4ub_31_. (*a*) Schematic diagram of the poly-nucleosome assembled on tandem repeats of the 208 base-pair Widom601 DNA. Nucleosome positions are represented by spheres and *Sca*I sites are indicated by dotted lines. (*b*) The unmodified, H2Bub_120_ and H4ub_31_ poly-nucleosomes were analysed by 0.7% agarose gel electrophoresis with EtBr staining. Lanes 1–3 indicate the unmodified, H2Bub_120_ and H4ub_31_ poly-nucleosomes, respectively. (*c*) Histone compositions of the unmodified, H2Bub_120_ and H4ub_31_ poly-nucleosomes were analysed by 18% SDS-PAGE with Coomassie Brilliant Blue staining. Lane 1 indicates molecular mass markers and lanes 2–4 represent the unmodified, H2Bub_120_ and H4ub_31_ poly-nucleosomes, respectively. (*d*) The *Sca*I digestion analysis. The unmodified, H2Bub_120_ and H4ub_31_ poly-nucleosomes were digested by *Sca*I, and the resulting mono-nucleosomes were fractionated by 5% native-PAGE with EtBr staining. Lane 1 indicates the naked DNA treated with *Sca*I. Lanes 2–4 represent the unmodified, H2Bub_120_ and H4ub_31_ poly-nucleosomes digested by *Sca*I, respectively. (*e*) Analytical ultracentrifugation sedimentation velocity analyses of the polynucleosomes. Sedimentation velocity analyses of the unmodified (open circles), H2Bub_120_ (open triangles) and H4ub_31_ (open diamonds) poly-nucleosomes were performed in the absence of MgCl_2_. The sedimentation coefficient (*S*_20,W_) distributions were calculated by the enhanced van Holde and Weischet method. (*f*) Sedimentation velocity analyses of the unmodified (closed circles), H2Bub_120_ (closed triangles) and H4ub_31_ (closed diamonds) poly-nucleosomes were performed in the presence of 1.25 mM MgCl_2_. The sedimentation coefficient (*S*_20,W_) distributions were calculated by the enhanced van Holde and Weischet method.

The H2B K120 monoubiquitination reportedly suppresses chromatin compaction [31]. To test whether the H4 monoubiquitination also affects chromatin compaction, we reconstituted poly-nucleosomes with H2Bub_120_ or H4ub_31_ (figure 4*a*). Twelve nucleosomes were assembled on tandem repeats of 208 base-pair 601 DNAs (figure 4*a*). The H2Bub_120_ and H4ub_31_ poly-nucleosomes were both reconstituted as efficiently as the unmodified poly-nucleosome (figure 4*b*,*c*). The restriction enzyme (*Sca*I) digestion analysis confirmed that trace amounts of the nucleosome-free 601 DNA segments were detected (figure 4*d*). These results indicated that the H2Bub_120_ and H4ub_31_ poly-nucleosomes were properly reconstituted.

We then performed a sedimentation velocity analysis by analytical ultracentrifugation [36]. Consistent with a previous report [31], the H2Bub_120_ poly-nucleosome exhibited sedimentation values of about 30*S*, similar to the unmodified poly-nucleosome in the absence of Mg^2+^ ion (figure 4*e*), but migrated more slowly than the unmodified poly-nucleosome in the presence of MgCl_2_ (1.25 mM) (figure 4*f*). These results indicate that the H2B monoubiquitination reproducibly suppressed the Mg^2+^-dependent chromatin compaction. We then tested whether the H4 monoubiquitination at position 31 affects chromatin compaction, because like the H2B monoubiquitination, it may also function in transcription activation [26]. Our sedimentation velocity analysis revealed that the H4ub_31_ poly-nucleosome also exhibited slow sedimentation, similar to that of the H2Bub_120_ poly-nucleosome, in the presence of Mg^2+^ ion (figure 4*f*). The sedimentation values of the H4ub_31_ poly-nucleosome were indistinguishable from those of the unmodified and H2Bub_120_ poly-nucleosomes in the absence of Mg^2+^ ion (figure 4*e*). These results indicated that the monoubiquitination at position 31 of H4 antagonizes chromatin compaction, similar to the H2B K120 monoubiqitination.

## 3. Discussion

Monoubiquitination of core histones has been identified as a major histone modification [11–14,22]. Substantial amounts (about 1–5% for H2B) of core histones are monoubiquitinated in cells [22], suggesting that the contribution of histone monoubiquitinations in genome function may be important. In this study, we focused on the H2B and H4 monoubiquitinations, which are found in the transcriptionally active loci of genomes, and reconstituted nucleosomes with ubiquitin molecules conjugated at the H2B-120 and/or H4-31 positions.

We then performed biochemical and structural analyses. Monoubiquitinations of the H2B K120 and H4 K31 residues have been proposed to stimulate transcription [23,24,26,37,38]. Consistent with this idea, the poly-nucleosomes containing H2Bub_120_ or H4ub_31_ formed a more relaxed conformation, when compared with that of the unmodified poly-nucleosomes, under the physiological Mg^2+^ conditions (figure 4). These results suggested that the H2B K120 and H4 K31 monoubiquitinations may confer a relaxed chromatin formation that is favourable for transcription factor binding and RNA polymerase passage. However, we found that the impact on the nucleosome stability is different between the H2Bub_120_ and H4ub_31_ nucleosomes (figure 2), although the structure of the nucleosome core particle was not affected (figure 3).

Our thermal stability assay revealed that the stability of the H4ub_31_ nucleosome is lower than that of the unmodified nucleosome (figure 2). In the nucleosome structure, the H4 K31 residues are located close to the DNA (figure 3*c*) and may interact with the DNA backbone by water-mediated hydrogen bonding [4]. The H4 K31 monoubiquitination may disrupt these interactions and cause nucleosome instability. To our surprise, we found that the H2Bub_120_ nucleosome is more stable than the unmodified nucleosome (figure 2). Similar nucleosome stabilization by H2B K123 monoubiquitination in yeast has been reported [39]. In contrast with the K31 residue of H4, the H2B K120 residues are exposed to the solvent in the nucleosome (figure 3*c*) [3,4,40] and may not affect the histone–DNA interactions within the nucleosome.

A plausible explanation for the H2B monoubiquitination-mediated stabilization of the nucleosome is that the ubiquitin molecules conjugated to the nucleosomal H2B molecules may interact with the histones within the nucleosome. The acidic patch may be an interactive site for the ubiquitin. Although the ubiquitin molecule does not directly interact with the histone surface in the SAGA–DUB–nucleosome complex, the ubiquitin molecule conjugated to the H2B K120 residue is located in a position that can directly interact with the acidic patch of the nucleosome surface, in the absence of SAGA–DUB [35]. By contrast, the ubiquitin molecule conjugated to the H4 K31 residue may be too far away to directly interact with the acidic patch. Understanding the mechanism of nucleosome stabilization by the H2B monoubiquitination at position 120 is an important issue to be addressed next.

The different stabilities between the H2Bub_120_ and H4ub_31_ nucleosomes suggest the distinct roles of the H2B and H4 monoubiquitinations. For example, the H4 K31 monoubiquitination renders the nucleosome more displacable and may facilitate RNA polymerase passage through nucleosomal DNA in gene bodies. By contrast, the H2B K120 monoubiquitination may function as a mark for specific chromosome loci by its stable association. Given that these monoubiquitinations are incorporated into gene body regions, the different stabilities of the H2Bub_120_, H4ub_31_ and unmodified nucleosomes may regulate the velocity of the RNA polymerase passage, and thus may control RNA production. Further studies of genomic localizations, gene expression and chromosome dynamics will clarify how the monoubiquitinations of histones contribute to the control of genomic DNA function in cells.

## 4. Material and methods

### 4.1. Preparation of recombinant proteins

Human recombinant histones (H2A, H2B, H3.2 and H4) were purified by the method described previously [40]. The DNAs encoding the human histone H3.2 C110A, H2B K120C and H4 K31C mutants were inserted between the *Nde*I and *Bam*HI sites of the pET15b vector. Human recombinant histones H3.2 C110A, H2B K120C and H4 K31C were expressed in *Escherichia coli* cells and purified, as described previously [40].

### 4.2. Preparation of monoubiquitinated histones H2B and H4

Purified histone H2B K120C or H4 K31C was mixed with 2,2′-dithiobis(5-nitropyridine) (DTNP) and the sample was dialysed against sterile water. The C-terminally cysteamine-fused ubiquitin protein was produced as described previously, with minor modifications [32]. The DNA encoding human ubiquitin was inserted between the *Nde*I and *Sap*I sites of the pTXB1 vector. Ubiquitin was expressed in *E. coli* BL21 (DE3) cells as the C-terminally intein-CBD-fused protein. The ubiquitin-intein-CBD fusion protein was loaded on a chitin column (New England BioLabs). The ubiquitin peptide was cleaved from the intein-CBD portion by an incubation with cysteamine-dihydrochloride (Sigma-Aldrich) and was eluted from the chitin column. The resulting ubiquitin-cysteamine peptide, which has a C-terminal aminoethanethiol linker, was further purified by gel filtration chromatography on HiLoad 26/60 Superdex 75pg (GE Healthcare). The peak fractions were dialysed against sterile water and then lyophilized. To conjugate the ubiquitin molecule, DTNP-treated histones H2B K120C and H4 K31C were mixed with the ubiquitin-cysteamine peptide in the 1 M HEPES-NaOH buffer (pH 6.9) containing 6 M guanidine hydrochloride. The resulting H2Bub_120_ and H4ub_31_ samples were further purified on a MonoS column (GE Healthcare).

### 4.3. Preparation of DNAs

The palindromic 146 base-pair satellite DNA [3] was purified by the method described previously [41]. The dsDNA fragment containing twelve 208 base-pair Widom601 DNA sequence repeats was prepared by the method described previously [42]. The DNA concentrations are expressed as moles of nucleotides.

### 4.4. Nucleosome reconstitution

To reconstitute the nucleosomes containing histones H2Bub_120_ and/or H4ub_31_, histones H3 C110A, H4ub_31_ or (H4), H2A and H2Bub_120_ (or H2B) were mixed in 20 mM Tris-HCl buffer (pH 7.5), containing 1 mM EDTA and 7 M guanidine hydrochloride. The samples were dialysed against 20 mM Tris-HCl buffer (pH 7.5) containing 2 M NaCl and the resulting histone octamers were further purified by gel filtration chromatography on HiLoad 16/60 Superdex 200 (GE Healthcare). Nucleosomes containing H2Bub_120_ and/or H4ub_31_ were reconstituted with the palindromic 146 base-pair satellite DNA fragment by the salt dialysis method, as described previously [40]. The DNA fragments were mixed with histone octamers in 10 mM Tris-HCl buffer (pH 7.5), containing 2 M KCl and 1 mM EDTA. The KCl concentration was gradually decreased to 250 mM, using a peristaltic pump. Reconstituted nucleosomes were further purified by non-denaturing 6% acrylamide gel electrophoresis, using a Prep cell apparatus (Bio-Rad).

### 4.5. Thermal stability assay

The thermal stability assay was performed in a 20 µl reaction mixture, containing 20 mM Tris-HCl (pH 7.5), 100 mM NaCl, SYPRO Orange (5×) and the nucleosomes (0.225 µg), according to the method described previously [33,34]. The fluorescence signals were detected using a StepOnePlus Real-Time PCR unit (Applied Biosystems), with a temperature gradient from 26 to 95°C, in steps of 1°C min^−1^. Normalization of the fluorescence intensity was calculated as (F(T) – F(26°C))/(F(95°C) – F(26°C)), where F(T) is the fluorescence intensity at a particular temperature.

### 4.6. Crystallization and determination of the nucleosome structures

The nucleosome solution containing H2Bub_120_ and H4ub_31_ (the H2Bub_120_/H4ub_31_ nucleosome) was concentrated to 4–6 mg ml^−1^. The crystals of the H2Bub_120_/H4ub_31_ nucleosome were obtained by the hanging drop vapour diffusion method, after mixing equal volumes of the H2Bub_120_/H4ub_31_ nucleosome solution and the reservoir solution (90 mM Tris-HCl (pH 7.8), 3.6% PGA-LM, 25.2% PEG400 and 2–6% pentaerythritol ethoxylate (3/4 EO/OH)), at 20°C. Crystals were soaked in the cryoprotectant solution, containing 90 mM Tris-HCl (pH 7.8), 3.6% γ-polyglutamic acid LM (PGA-LM), 30.6% PEG400, 2–6% pentaerythritol ethoxylate (3/4 EO/OH) and 2.7% trehalose at 4°C, and were flash cooled in a stream of N2 gas (−180°C). The dataset was collected at the BL-1A beamline in the Photon Factory (Tsukuba, Japan). Diffraction data were integrated and scaled with the HKL2000 program [43]. The structure of the H2Bub_120_/H4ub_31_ nucleosome was solved by the molecular replacement method, using the PHASER program [44] with the H3.2 nucleosome structure (PDB ID: 3AV1) as the search model [45]. The initial model of the H2Bub_120_/H4ub_31_ nucleosome was iteratively refined, using the PHENIX program [46]. Manual model building was performed using the COOT program [47]. The Ramachandran plot for the final structure of the H2Bub_120_/H4ub_31_ nucleosome was assessed by the MolProbity program [48]. A summary of the data collection and refinement statistics is shown in table 1. All structural graphics were made using the PyMOL program (Schrödinger; http://www.pymol.org).

**Table 1.** Data collection and refinement statistics.

resolution range (Å)	50–3.33 (3.46–3.33)
space group	P3_2_
cell parameters	*a* = 100.419 Å, *b* = 100.419 Å *c* = 186.025 Å, *α* = 90° *β* = 90°, *γ* = 120°
total number of unique reflections	30 012
*R*_merge_ (%)^a^	7.0 (32.0)
completeness (%)	97.7 (92.8)
redundancy	6.2 (3.1)
*I*/*σ* (*I*)	11.4 (2.3)
refinement	
resolution (Å)	39.391–3.330
*R*_work_/*R*_free_ (%)^b^	20.11/26.25
r.m.s.d. bonds (Å)	0.012
r.m.s.d. angles (°)	1.496
Ramachandran plot	
most favoured (%)	94.88
allowed (%)	5.12
disallowed (%)	0
PDB code	5B40

^a^*R*_merge_ = Σ*_hkl_*Σ*_i_*|*I_i_*(*hkl*) − 〈*I*(*hkl*)〉|/Σ*_hkl_*Σ*_i_I_i_*(*hkl*).

^b^*R*_work_ = Σ*_hkl_*||*F*_obs_| − |*F*_calc_||/Σ*_hkl_*|*F*_obs_|. *R*_free_ was calculated with 5% of the data excluded from the refinement.

### 4.7. Preparation of poly-nucleosomes

The poly-nucleosomes for the sedimentation velocity analysis were reconstituted with the histone octamer and the dsDNA containing twelve 208 base-pair Widom601 DNA sequence repeats (histone octamer/Widom601 sequence ratio = 1.8), as described previously [49]. Briefly, the DNA was mixed with the histone octamer in a 2 M NaCl solution, containing 10 mM Tris-HCl (pH 7.5) and 1 mM EDTA. The NaCl concentration was gradually decreased to 250 mM, using a peristaltic pump. The reconstituted poly-nucleosomes were further purified by non-denaturing agarose-acrylamide composite gel (0.7% agarose and 2% acrylamide) electrophoresis, using a Prep cell apparatus (Bio-Rad).

### 4.8. Analytical ultracentrifugation analysis

The poly-nucleosomes reconstituted on the 12 Widom601 sequence repeats were dialysed against 10 mM Tris-HCl (pH 7.5) buffer, in the absence or presence of 1.25 mM MgCl_2_. Sedimentation velocity analyses were performed with a ProteomeLab XL-I analytical centrifuge (Beckman Coulter). The samples (OD_260_ = 0.6–0.8) were incubated for 2 h at 20°C under vacuum conditions and were then centrifuged at 22 000 rpm in 12 mm double-sector cells. Collected data were analysed by the enhanced van Holde–Weischet method, using UltraScanII 9.9 revision 1927 [50]. Sedimentation coefficients (*S*_20,W_) were calculated with a partial specific volume of 0.65 ml g^−1^ [36].

### 4.9. *Sca*I analysis

The *Sca*I analysis was performed by the method reported previously [42,49]. The poly-nucleosomes (30 µM) reconstituted on the 12 Widom601 sequence repeats were treated with *Sca*I (14 units) in a reaction solution, containing 15 mM Tris-HCl (pH 7.5), 55 mM NaCl, 1 mM dithiothreitol, 100 µg ml^−1^ BSA, 5% glycerol and 0.5 mM MgCl_2_. After incubation at 22°C for 12 h, the samples were analysed by non-denaturing 5% acrylamide gel electrophoresis with EtBr staining.

## Data accessibility

PDB IDs: the atomic coordinates of the nucleosome containing H2B and H4 monoubiquitinations have been deposited in the Protein Data Bank, with the ID code 5B40.
